# Doxycycline reduces osteopenia in female rats

**DOI:** 10.1038/s41598-019-51702-y

**Published:** 2019-10-25

**Authors:** Fellipe A. T. de Figueiredo, Roberta C. Shimano, Edilson Ervolino, Dimitrius L. Pitol, Raquel F. Gerlach, Joao Paulo M. Issa

**Affiliations:** 1 Department of Biomechanics, Medicine and Rehabilitation of the Locomotor System, Ribeirao Preto Medical School, University of Sao Paulo – Av. Dos Bandeirantes 3900, Ribeirao Preto, SP CEP 14049-900 Brazil; 2 Department of Basic Sciences, Sao Paulo State University Júlio de Mesquita Filho – R. Jose Bonifácio 1193, Araçatuba, SP CEP: 16015-050 Brazil; 3 Department of Basic and Oral Biology, School of Dentistry of Ribeirao Preto, University of Sao Paulo – Av. Cafe S/N, Ribeirao Preto, SP CEP: 14040-904 Brazil

**Keywords:** Osteoporosis, Preclinical research

## Abstract

Doxycycline, a member of the tetracycline family, is a drug used as an antibiotic (dosage of 100 mg/day) and as an anti-inflammatory drug on the dosage of 20 mg twice a day, this use has Matrix Metalloproteinases (MMP) inhibitor action. Doxycycline is a calcium chelator and therefore interferes in bone remodeling. The main objective of this study was to evaluate the action of the drug doxycycline in the control of osteopenia. Sixty three Wistars rats were divided into 9 groups with n = 7 each, as follow: the control group with doxycycline 10 mg/kg/day (C10), control with doxycycline 30 mg/kg/day (C30) and control (C), ovariectomized group with doxycycline 10 mg/kg/day (OVX10), ovariectomized with doxycycline 30 mg/kg/day (OVX30), and ovariectomized with water (OVX), sedentary group with 10 mg/kg/day (Se10), sedentary with doxycycline 30 mg/kg/day (Se30), and sedentary group with water (Se). Left femoral bone was used for bone densitometry, right femoral bone for histological analysis. The right tibia was intended for chemical quantifications, the total serum was used for cholesterol and calcium quantification. The length of the left femoral bone was measured after the densitometry analysis. Statistical analysis was performed using multivariate general linear model (ANOVA two factors with Bonferroni adjustment) and the TRAP analysis was subjected to normality test and then were subjected to nonparametric test, both with p < 0.05 significance. Statistically significant differences were found, with better results for the groups exposed to the medication (10 and 30 mg/kg/day): Se vs. Se10 and Se vs. Se30 for BMC, quantification of magnesium, amount of cancellous bone in the distal portion; OVX vs. OVX10 for BMC, BMD and calcium in serum; OVX vs. OVX10 and OVX30 for quantification in proximal and distal portion of cancellous bone; Se vs. Se30 and OVX vs. OVX30 for immunostaining for TRAP, all results with minimum of p ≤ 0.05. Doxycycline had a deleterious effect on control groups and positive action for bone organization on female rats affected by bilateral ovariectomy-induced osteopenia and sedentary lifestyle.

## Introduction

## The Role of Doxycycline

**Figure 1:**
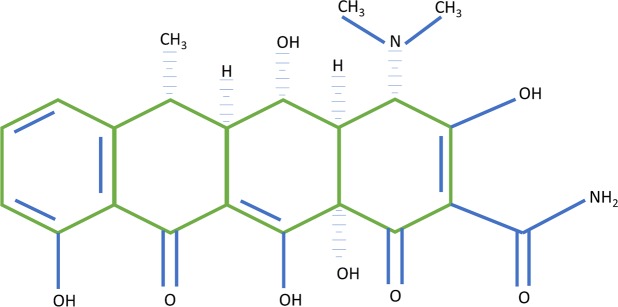
Representation of the chemical structure of Doxycycline.

Doxycycline is a member of the tetracycline family, a drug used as an antibiotic (dosage of 100 mg/day) and as matrix Metalloproteinases (MMPs) inhibitor (dosage of 20 mg twice a day), which use has an anti-inflammatory effect. The molecule of tetracycline is defined as naphthacene carboxamide ring system^1^, Doxycycline differs structurally from the tetracycline molecule in the modifications of substituent at positions 5 and 6 of the ring system, which make it more lipid soluble and could be presented in the anhydrous form (Fig. 1)^2^. Doxycycline on this low-dose is considered for the treatment of chronic periodontitis^3^ and chronic inflammation in the skin^4^. For these uses doxycycline in “subdoses” (Subantimicrobial dose doxycycline or SDD) was approved in 1998 by the Food and Drug Administration (FDA) in the USA and also by the regulatory agencies of Canada and Europe. These formulations of doxycycline are named Periostat and Oracea (in the US) and the use can be done continuously for 9 months. This dose does not induce microbial resistance^5^.

## Doxycycline and Bone Interactions

Doxycycline interacts with bone and teeth, accumulating on these tissues during their mineralization (teeth and bone) and during the physiologic remodeling (bone)^6^. In bone metastasis from breast cancer and prostate cancer, doxycycline has a curious role on the bone remodeling inducing the bone formation^7^ and inhibiting the MMP associated to this tumor^8^. SDD of this medication is related to reduction of serum biomarker of bone resorption and related to the increase of biomarker of bone formation^9^ in postmenopausal osteopenic women with 2 years follow-up. These interaction with the bone are not related to the antimicrobial interaction (SDD), but doxycycline can act as inhibitory inflammatory medication of the bone resorption^10^.

## Osteoporosis, Public Health and Induction of Osteopenia

Osteoporosis is a classical disorder of elderlies, responsible for increased risk of bone fractures^11^, characterized as an increase of skeletal fragility^12^. Osteopenia is defined as “low bone mass” and it is a warning for the future osteoporosis. Exercises and a better quality of life might prevent the fracture of the bones affects by this low mass^13^.

The incidence of fractures due to osteoporosis is increasing and it also increases the costs of public health to treat this disorder^14,15^. This information is important because it has been shown in study that during the youth practicing physical exercises and balanced diet are crucial for prevention of osteoporosis. Also during childhood and adolescence, it is essential for the increase of bone density and bone growth in humans^16^.

Bone remodeling depends on a balance between bone resorption and bone formation^17^. Considering the high prevalence of osteoporosis in many populations, researchers have proposed animal models for osteopenia induction. One of them is the ovariectomy-induced menopause that mimics the increase in osteoclastic activity which exceeds the osteoblastic activity, resulting an induction of osteoporosis^18^. Rats are the animal model used for this purpose^19^ because they supply enough sample for statistical purposes, easy feed and location. Different research groups worked with this type of osteopenia induction in Wistar rats^20–24^.

## Sedentarism and Osteopenia

Nowadays young people and children are being “victims” of modern life sedentarism and obesity^25,26^, exactly during this time of life, prevention of chronic diseases of adult life needs to be initiated (high blood pressure, dyslipidemias, diabetes mellitus)^16,27^. Moreover, it is pointed out that the practice of exercises in the youth, when the bones are more malleable, promotes better bone quality in the future^28–31^. According to NiA (National institute of age, NIH, USA), weight training, walking, hiking, jogging, climbing stairs, tennis, and dancing can prevent the fractures of bones affected by this low bone mass. Karlsson and collaborators studied about football players who have left their profession for over 20 years, demonstrating a residual protective effect on bone quality after the long time without performing intense exercises^32^, characterizing that sports at early age contribute to a better quality of life in elderly phase.

A calcium deficient diet, common on sedentary diets, also induces osteopenia as demonstrated by a meta-analysis Specker (1996), who presented a result showing that a sedentary lifestyle (no exercise) combined with a calcium-poor diet worsens the picture of osteopenia, as shown by data obtained from bone densitometry^33^.

With all of this important knowledge of osteopenia and it is relation with the drug doxycycline, the main objective of this study was to evaluate the action of the drug doxycycline in the control of osteopenia induced by bilateral ovariectomy and sedentary lifestyle in Wistar female rats.

## Materials and Methods

**Figure 2:**
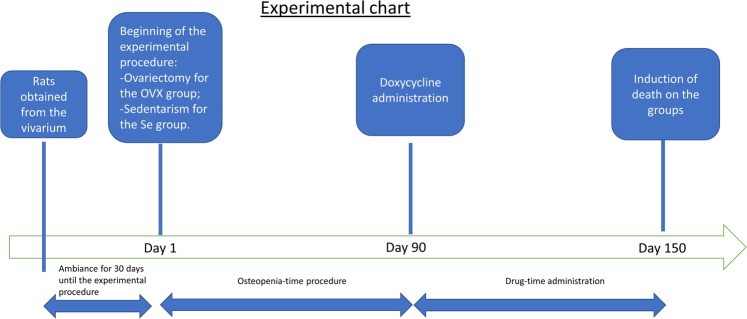
Flow chart with the interventions on present work.

This study was approved by the Ethics Committee on use of animals in research of the University of Sao Paulo: School of dentistry of Ribeirao Preto (in Portuguese CEUA/FORP-USP) under the Protocol of 2013.1.1528.58.5 in confirmation to all the methods and guidelines of CONCEA (National guideline of animal use on experimentation from Brazil), which follow the international parameters on use and handling of animals. Sixty three (63) female Wistar rats with 45 days of life (or 100 g) were obtained from the campus of the University of São Paulo, Ribeirao Preto and stayed at the School of Dentistry of Ribeirao Preto for ambiance for 30 days until methodological procedure. Experimental procedures of groups were started with 75 days of age (or 150 g), this age has been considered as day1 of experimental time (Fig. 2). Wistar females reached sexual maturity at this age and it is known that bone maturity is acquired only by 10 months of age (or 300 days of life)^19^. The doxycycline administration starts on day 90 of treatment (or 165 days of life) and it was administered for 60 days until the induction of death.

### As for the division of the groups

Control group with Doxycycline 10 mg/kg/day (C10), Control with Doxycycline 30 mg/kg/day (C30), and Control (C), Ovariectomizated Group with Doxycycline 10 mg/kg/day (OVX10), Ovariectomizated with Doxycycline 30 mg/kg/day (OVX30) and Ovariectomizated with water (OVX), Sedentary group with 10 mg/kg/day (Se10), Sedentary group with 30 mg/kg/day (Se30) and Sedentary group with Water (Se).

### Procedure of ovariectomy

The animals were anesthetized with Xylazine and Ketamine in concentration 10 mg/kg and 100 mg/kg, respectively, followed by trichotomy and the skin were prepared with antiseptic. A caudal midline incision was made from the umbilicus to the pelvis and the ovarian horn was exteriorized between the bottom of costal edge and the top of the pelvis. Shortly thereafter the evisceration of the ovary horn was executed followed by ovary ligation for containing the bleeding. The same procedure was performed on the opposite side to the excision to excise both ovaries^20,22,23^. After the surgical procedure, the application of antibiotics was performed (Veterinary pentabiotic, Zoetis, Parsippany-Troy Hills, New Jersey, USA) once a day for a period of 48 hours after the surgery. The osteopenia was induced for 90 days after the surgical procedure, once significant findings occurring through this time of experiment^34,35^. Then the groups received doxycycline (Bioquanti, compounding pharmacy, Ribeirao Preto, SP, Brazil) at 2 concentrations for 60 days.

### Procedures for the sedentary groups

Females of this group were divided in individual cages with 30cmx20cmx16cm with superior grid, limiting their movements for 90 days to mimic a sedentary lifestyle^24,36,37^. Then the Se10 and Se30 groups received the medicine for 60 days. The low locomotion induction of osteopenia (Sedentary lifestyle induction on rats) is used not as a method of disuse, but a method of restriction of movement, that culminates on osteopenia.

### Administration of doxycycline

**Figure 3:**
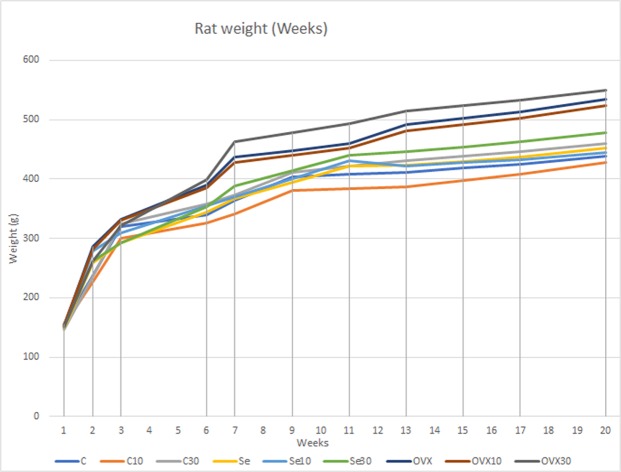
Graph of the weights of the rats from day of experiment until the day of induction of death (20th week).

After the 90 days, doxycycline was administered for 60 days (for comparison between groups) in drinking water^22^, serviced according to a daily chart daily chart of total body weight (Fig. 3) and based on how much the rats drank water, with all duly noted and monitored. The two chosen doses were based on previously published studies^38–41^, their application were assed because of it is safe use for 60 days and for the mimetics aspect with the SDD use in humans for 2 years.

At the end of the experiment, the animals were euthanized by an overdose of anesthetic (xylazine at 30 mg/kg and ketamine at 150 mg/kg) followed by CO_2_ inhalation. The bones were removed and cleaned of soft tissue for the subsequent analyses.

### Sampling and analysis

The left femoral bone samples were intended for bone densitometry and samples of right femoral bone were intended for histological analysis. It has been used samples of right tibia for chemical quantification of calcium, magnesium, zinc and phosphorus. The total weight of the rats was assessed in precision scales. The left femur (dry by an oven at 37 degrees Celsius) was weighted in laboratory precision scale BioPrecisa FA2104N (Curitiba, PR, Brazil) and the length of the femoral bones were measured with the aid of a digital caliper (MTX group, Guarulhos, Sao Paulo, Brazil) that measured the bones in your long axis. The blood serum was used for measurement of calcium and total cholesterol.

### Quantification of calcium and cholesterol in serum

The whole blood of animals was obtained by cardiac puncture. Tubes containing the whole blood were centrifuged (800 × *g*, 6 min) to obtain serum^42,43^. Syringes and needles were previously decontaminated in nitric acid 3%^44^.

For calcium measurement, the catalyst of phosphorus (phosphate binder on medical activities^45–47^) content of sample was the lanthanum that separate this typical interference of this sample in this kind of analysis^48^. Lanthanum was placed on ratio of 0.5% of the total volume thinned and the samples were analyzed in a flame atomic absorption (Perkin Elmer AAS400, Norwalk, USA).

Quantification of total cholesterol was perform using the Cholesterol Liquiform Kit (Labtest Diagnóstica, Lagoa Santa, MG, Brazil) in a colorimetric plate adapted for the BioTek μQuant (BioTek, Winooski, VT, USA) equipment. The method used for this measurement was “End-point Colorimetric Assay - 500 nm”.

### Bone densitometry

**Figure 4:**
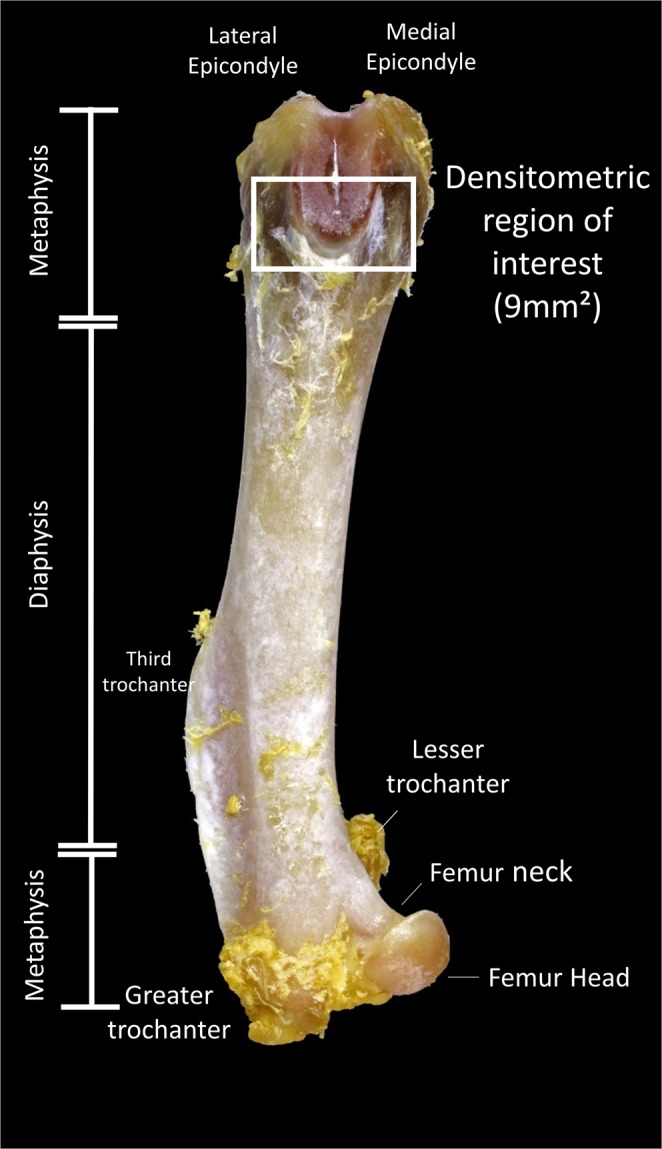
Photograph of a femur bone used on the present work showing the area of interest for the densitometric analysis (Square symbol on the photograph) and the anatomical points distinguishing the area of interest.

The left femora were submitted to densitometric analysis using “dual-energy” x-ray densitometer equipment, Alpha, Lunar DPX ® (Lunar, Madison, WI, United States) installed at the School of Medicine of Ribeirao Preto. Adapted for measurement small animals with the PIXImus software (Lunar, Madison, WI, United States)^49,50^. The image acquisition is proceeded with the femurs (distal portion of the femur) position with ROI of 9 mm² (Fig. 4), immersed in a depth of 2 cm of deionized water (simulating soft tissues) and the following options have been selected: Type 1 Appendicular Bone; high resolution mode; 76 kVp; 150 μA; Collimation; standard 40 mm × 20 mm areas.

Bone mass content (BMC) and bone mineral density (BMD) were obtained.

### Chemical analyses of calcium, magnesium and zinc in the bones

All materials used in the analysis were decontaminated in distillated nitric acid. To perform the analytical test a bi-distillated acid was diluted with deionized water ultrapure to reach 3% of acid on solution. Bone was dried in Chapel of laminar flow, to prevent the deposition of dust^44^. All this was done to avoid contamination of specimens.

The right tibias were submerged in half on bi-distilled nitric acid 3% for 1 minute^51^, then the bone was withdraw and the solution were submitted to chemical analysis. The biopsy was performed to make the results measurable on the calibration curve.

### Immunohistochemistry analysis by TRAP

**Figure 5:**
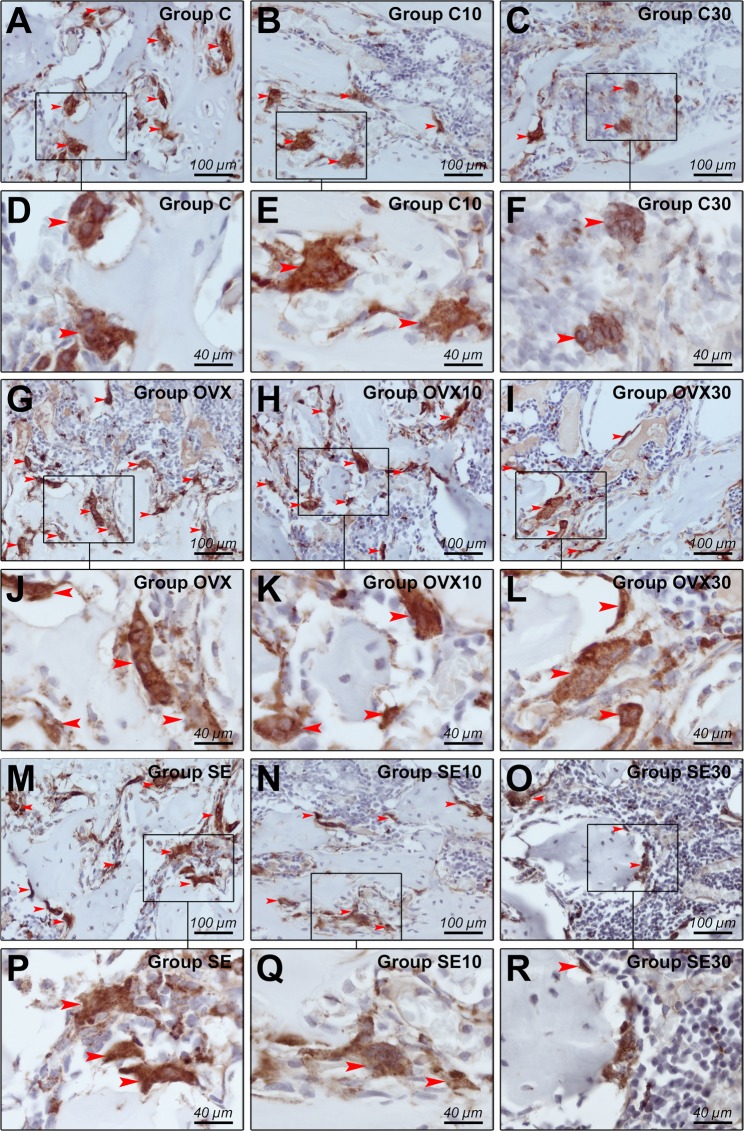
Immunolabeling for TRAP. (**A**–**R**) Photomicrography’s showing the immunolabeling standard for TRAP in groups C (**A**,**D**), C10 (**B**,**E**), C30 (**C**,**F**), OVX (**G**,**J**), OVX10 (**H**,**K**), OVX30 (**I**,**L**), Se(**M**,**P**), Se10 (**N**,**Q**), Se30 (**O**,**R**). Symbols: Red arrows: Immunolabeling cells, and rectangles: increases in the areas listed. Counter-staining: Harris Hematoxylin. Increase: (**A**–**C**), (**G**–**I**), (**M**–**O**) 400x of magnification; (**D**–**F**), (**J**–**L**), (**P**–**R**) 1000x of magnification.

The immunohistochemical analysis was performed by a certified morphologist and blind to the treatment. A semi-quantitative analysis was carried using a histological section of each animal, in the original increase of 200X. The pattern of immunolabeling was assigned a score. The criterion adopted for the establishment of the scores was based and modified from those established by Faria *et al*.^52^, where: 0 SCORE = absence of immunolabeling; 1 = low SCORE ± standard; 2 = moderate SCORE ± standard; 3 = high SCORE ± standard (Table 1 and Fig. 5).

**Table 1:** Immunolabeling for TRAP.

TRAP (ESCORE – MEDIAN)		
C	**SCORE 2**	**MODERATED** immunolabeling
C10	**SCORE 1**	**LOW** Immunolabeling
C30	**SCORE 1**	**LOW** Immunolabeling
OVX	**SCORE 3** ^**a**^	**HIGH** Immunolabeling
OVX10	**SCORE 2**	**MODERATED** Immunolabeling
0VX30	**SCORE 1**	**LOW** Immunolabeling
Se	**SCORE 3** ^**b**^	**HIGH** Immunolabeling
Se10	**SCORE 2**	**MODERATED** Immunolabeling
Se30	**SCORE 1**	**LOW** Immunolabeling

**A-i:** photomicrography showing the standard to TRAP in C (**A**), C10 (**B**), C30 (**C**), (**D**) OVX, OVX10 (**E**), OVX30 (**F**), Se (**G**), Se10 (**H**), Se30(**I**). ^a^Indicates the difference between OVX *vs*. OVX30 and ^b^indicates difference between Se and Se30 with p < 0.00001 between the groups with the same letter superscript. Symbols: Red arrows: immunostained cells. Against-coloration: Harris Hematoxylin. Magnification: 1000x. Scale bars: 30 micrometers.

### Analysis of bone by masson trichrome and picrosirius-red staining

**Figure 6:**
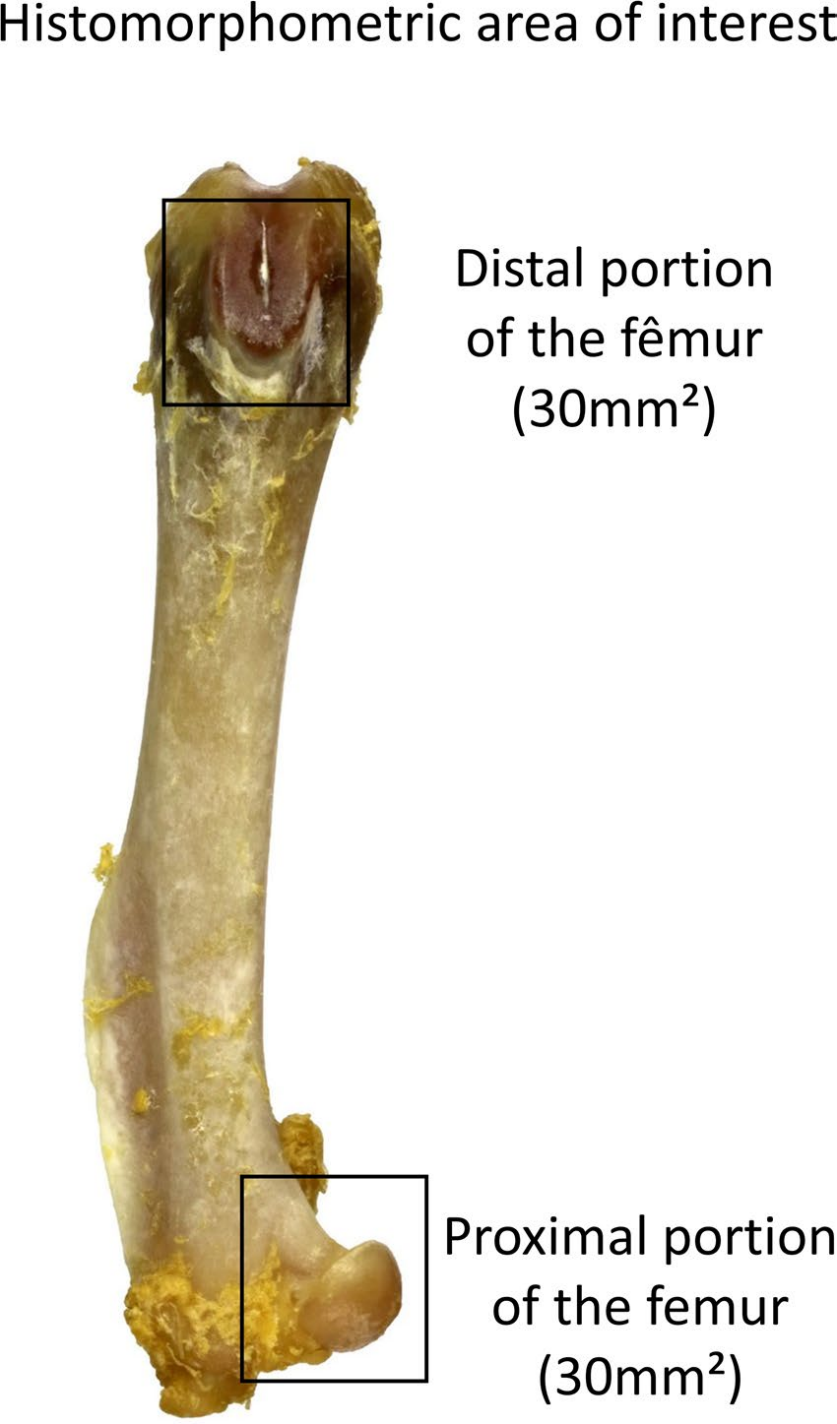
Photograph of a femur bone used on the present work showing the area of interest used for the quantification of cancellous bone (Square symbols on the photograph).

**Figure 7:**
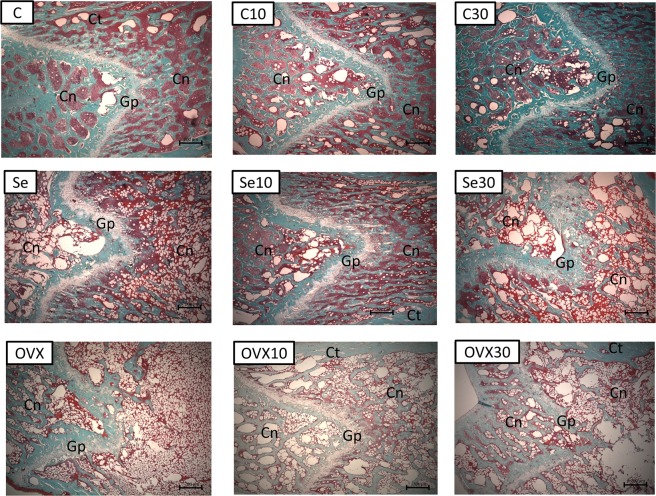
Histological photomicrograph of femur bone at distal portion, demonstrating the cancellous bone (Cn), growth plate (Gp) and cortical bone (Ct). Staining: Masson trichrome: 25x of magnification.

**Figure 8:**
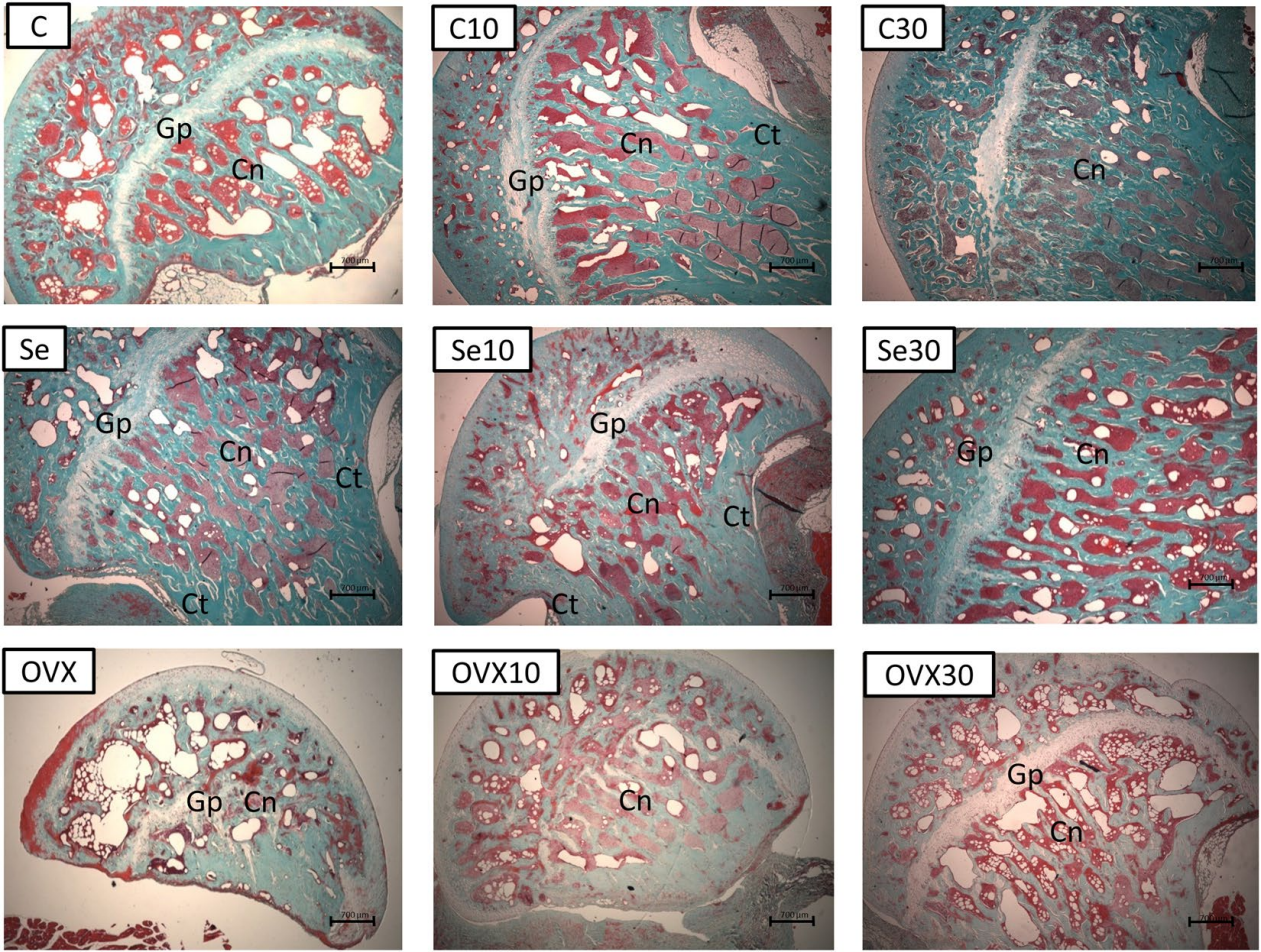
Histological photomicrograph of femur bone at proximal portion, demonstrating the cancellous bone (Cn), growth plate (Gp) and cortical bone (Ct). Staining: Masson trichrome: 25x of magnification.

The quantitative analysis of the histological glasses allows to evaluate the bone microarchitecture in the proximal and distal epiphysis areas by Masson trichrome staining^22,23^. Picrosirius-red staining allows to quantify the total collagen on the histological glasses. It was used a light microscope Axioimager Z2 Zeiss (Carl Zeiss, Oberkochen, Germany) coupled to a digital camera, aided by a DELL Precision T3500 workstation (DELL, São Paulo, Brazil). The digital images obtained from the Masson trichrome staining were analyzed by the software AxioVision 4.8 (Carl Zeiss, Oberkochen, Germany) on 25x of magnification. The quantification of total collagen obtained from picrosirius-red staining was obtained on magnification of 40x and was analyzed by ImageJ program (NIH, USA) using the plugins needed for this achievement. For quantitative analysis were focused the area of 30 mm² of the cancellous bone region (withdraw the growth plate), a distal and proximal acquisitions (Fig. 6), histological sections with 5 µm of thickness stained by Masson trichrome were analyzed by quantitative and qualitative analysis between the groups and were discussed below in the section “Results”^53^ (Figs 7 and 8).

### Analysis of the samples

Densitometry results were demonstrated in mg/mm^2^ for BMD and Milligram (mg) for BMC with the x-rays focusing on 9 mm^2^ on distal portion of femoral bone. Quantification data from calcium, magnesium and zinc were expressed in milligram per gram (mg/g). Quantification of cancellous bone was expressed on Pixels². Measures of total weight of animals were expressed in grams (g). Femur length was shown in centimeters (cm) and the bone weight was demonstrated in milligrams (mg). Serum samples were subjected to measurements of the quantity of total calcium and total cholesterol, these data were expressed in milligram per deciliter (mg/dL).

### Statistical analysis

The software used for statistical analysis was SPSS 20.0 (IBM, New York, NY, USA) and the samples were submitted to the general linear model test, and all comparisons were submitted to Bonferroni adjustment^24,49^. Analysis of TRAP were subjected to normality test and then it was subjected to nonparametric test with Kruskal Wallis as analysis of variance, with a post-test of Dunn that was held to specify these differences. The results were considered different when p ≤ 0.05.

## Results

The results represented on the Table 2 have the first column with the analysis made. The other 3 columns have the results mean (standard deviation) and the fourth column have the p value with the differences points as “^a^” and “^b^” corresponding to the line of the analysis.

**Table 2:** Comparison between treatment groups considering only significant differences (p ≤ 0.05).

Type of analysis\Groups	C	C10	C30	p Value	Se	Se10	Se30	p Value	OVX	OX10	OVX30	p Value
**Results compared between the groups: Mean (Standard deviation) - p** **≤** **0**.**05**												
BMC (mg)	26.7 (5.9)^a.b^	21.8 (3.9)	18.6 (2.5)	^a^p = 0.018 ^b^p < 0.0001	12.7 (2.5)^a.b^	21.3 (2.3)	20.4 (1.6)	^a^p < 0.0001 ^b^p < 0.001	8.8 (3.4)^a^	13.1 (1.3)	10.8 (2.3)	^a^p = 0.044
BMD (mg/mm²)	2.8 (0.6)	2.4 (0.4)	2 (0.2)		1.4 (0.2)	2.3 (0.2)	2.2 (0.2)		0.9 (0.4)^a^	1.4 (0.1)	1.2 (0.2)	^a^p = 0.031
Calcium Bone Biopsy -mg/g	114.2 (8.2)^a.b^	38 (5.5)	27 (8.5)	^a^p < 0.001 ^b^p < 0.001	27.1 (9.2)	32.5 (4.3)	32.9 (6.9)		48 (9.9)	54.2 (10.5)	54.3 (12.2)	
Magnesium Bone Biopsy – mg/g	10.5(2.8)^a.b^	4.2(0.7)	4(0.9)	^a^p < 0.001 ^b^p < 0.001	3.4(1.4)	4.9(0. 7)	7.8(2.7)^a.b^	^a^p < 0.001 ^b^p = 0.007	6.2(1.6)	6.3(1.4)	6.3(1.3)	
Zinc Bone Biopsy -mg/g	0.7(0.2)^a.b^	0.2 (0.1)	0.2(0.06)	^a^p < 0.001 ^b^p < 0.001	0.2(0.1)	0.3(0.1)	0.5(0.1)		0.4(0.08)	0.4(0.06)	0.4(0.1)	
Calcium on serum – mg/dL	12.87(23.5)	11.09(11.1)	11.84(38.6)		14.04(13.9)	14.14(20.3)	13.10(47.3)		9.36(89)^a^	12.22(21.6)	13.75(36.5)	^a^p = 0.013
Total Cholesterol on serum – mg/dL	100(14.5)	103(15.5)	88(15.5)		115(17.7)	110(15.8)	94.7(15)		122.7(15.8)	164(17)	126(17)	
Total collagen on proximal portion of femur (%)	13.4(3.9)	16.6(4.2)	16.6(4.1)		21.9(4.2)	14.8(4.2)	20.9(4.3)		12.7(4.3)	12.2(4.7)	13.3(4.8)	
Total collagen on distal portion of femur (%)	13.3(3.4)	9.6(3.7)	11.7(3.7)		11.6(4.2)	15.3(3.7)	11.1(3.3)		9(3.7)	12.2(4.2)	19.4(4.1)	
Quantification of cancellous bone on proximal portion of femur (10^−5^) Pixel^2^	15.5(1.6)	14.9(1.8)	19.6(1.7)		15.9(2)	15(1.8)	16.9(1.6)		8.7(1.8)^a.b^	11.7(2)	17.6(2)	^a^p = 0.04 ^b^p = 0.05
Quantification of cancellous bone on distal portion of femur (10^−5^) Pixel^2^	20.3(1.3)^a^	16.1 (1.5)	13.9 (1.4)	^a^p = 0.036	9.4 (1.7)^a.b^	14.7 (1.5)	17.4 (1)	^a^p = 0.05 ^b^p = 0.02	9.2 (1.5)^a.b^	15.1 (1.7)	18.0 (1.5)	^a^p < 0.01 ^b^p = 0.03
Femur Height – mm	40.1(1.2)	36.9 (3.6)	38.4(2.9)		38.6(1.6)	37.6 (3)	37.2(4.2)		37.9 (2.4)	36.9 (4.2)	39.1 (2.6)	
Total weight - g	438.4 (43.5)	428 (44.1)	460.4 (30.1)		451.4 30.2)	444 (34)	478 (33.2)		535 (26)	523.1 (41.1)	550.1 (39.1)	
Dry weight of femur bone- mg	731.2 (102.6)	684 (92.2)	782.7 (76.9)		791.8 (155.5)	850.7 (161.1)	851.1 (152.5)		885.4 (61)	827.8 (108.3)	872.1 (95.4)	

The value of p is shown on the column “p Value” with “^a^” and “^b^” superscript showing the differences, and if is not different it was shown no superscript letter.

### Preclinical findings

Twenty eight percent of the rats from Se group (because they were isolated in individual cages) presented eruptions on skin at the final week of the study, as a result of absence hygiene, motivated by the absence of contact with other animal of the same species. Due to this, it was managed a daily treatment with rifamycin (Rifocina Spray, Sanovi Aventis, Sao Paulo, Brazil) until the induction of death and it was guaranteed that the use of this medicine did not influence on the results of this study, according to studies of bone repair where it look for infections prevention of the surgical region^54^. All the animals were on good health and well treated before the palliative treatment with rifamycin.

### Quantitative analysis

Quantification of cancellous bone by Masson trichrome was expressed in Pixel^2^ ^22,23^, quantification by picrosirius was expressed as percentage of total collagen (%)^55^ and immunolabeling for TRAP was expressed by Scores^21^.

The immunohistochemical technique for TRAP (Tartrate-resistant acid phosphatase) showed high specificity in detecting such protein. This specificity indicates an immunolabeling of osteoclastic activity^56^, which was proven by the total absence of markup in the negative control reaction. Immunoreactive cells showed a brownish staining confined exclusively to the cytoplasm. The differences found was Se *vs*. Se30 and OVX *vs*. OVX30 with p < 0.00001, been lower osteoclast expression on OVX30 and Se30, compared to control OVX and Se, data shown on Table 1.

Table 2 shows the values of the averages with their respective standard deviations in parentheses and followed by the p value when there are significant differences, showing a comparison between groups.

BMC demonstrated that the control C vs. C10 (p = 0.018) and C *vs*. C30 (p < 0.0001) had statistically significant differences, been higher values of BMC attributed to the control C. In the OVX group, OVX *vs*. OVX10 (p = 0.044) had significant differences. The group Se *vs*. Se10 (p < 0.0001) and Se *vs*. Se30 (p < 0.001) had significant differences with higher values attributed to the groups treated with doxycycline.

BMD for OVX group showed the following difference, OVX *vs*. OVX10 (p = 0.031). Sedentary group presented Se *vs*. Se10 (p < 0.001) and Se vs. Se30 (p < 0.001) with higher values attributed to the groups treated with doxycycline. No difference was found between C, C10 and C30.

For calcium quantification on periosteal surface of distal half of tibia bone biopsy, it was observed in the control group C *vs*. C10 (p < 0.001) and C *vs*. C30 (p < 0.001) had statistically significant differences with higher values of calcium concentration on the control C. Groups OVX and Se had no statistically significant, but have a tendency of increase of this alkaline earth metal on the groups exposed to doxycycline.

For magnesium and zinc measurements on periosteal surface of distal half of tibia bone biopsy, control group C *vs*. C10 (p < 0.001) and C *vs*. C30 (p < 0.001) had statistically significant differences with higher values of magnesium and zinc concentration on the control C. In the OVX group was not obtained significant differences on both analysis, but for quantification of magnesium on the Se group, it was observed differences when it compares the Groups Se *vs*. Se30 (p < 0.001) and Se *vs*. Se10 (p = 0.007) with higher values on the groups exposed to the doxycycline (Se10 and Se30).

Quantification of calcium on serum of control and sedentary groups, no significant differences were found, but for OVX group it was observed that OVX *vs*. OVX30 (p = 0.013) had statistically significant differences with higher concentration of calcium on serum on the group OVX30 and a tendency of increase dose-dependent of doxycycline medication on this particular group.

Quantification of cancellous bone by Masson trichrome on proximal portion of femur bone had OVX *vs*. OVX10 (p = 0.04) and OVX *vs*. OVX30 (p = 0.05) with statistical differences with an increase of cancellous bone on the groups treated with doxycycline.

Quantification of cancellous bone by Masson trichrome in distal portion of femur bone region had for the control group (C) *vs*. C30 (p = 0.036) significant differences. Ovariectomized group had OVX *vs*. OVX10 (p < 0.01) and OVX *vs*. OVX30 (p = 0.03) significant differences. Sedentarism group presented the following statistical differences, Se *vs*. Se10 (p = 0.05) and Se *vs*. Se30 (p = 0.02). All the results of cancellous bone on distal portion of the bone showed that doxycycline took by control group were no beneficial, so the C has higher values than C30, but when the disease is established, the medication showed higher values for Se10 and S30 and OVX10 and OVX30 in comparison to it is controls.

On the other hand, for total weight of the rats before death induction, total cholesterol, quantification of collagen by picrosirius, total length of femurs and femoral weight, no differences were found.

### Qualitative analysis for histology

#### Trichrome of masson

Histological photomicrographs stained by Trichrome of Masson are represented at distal portion – Fig. 7 and proximal portion – Fig. 8. The cancellous bone (Cn), growth plate (Gp) and the cortical bone (Ct) are indicated in both figures.

The letters in the upper left corner are referred to the experimental groups of this work Group C: control without doxycycline or any treatments; Group C10: Doxycycline 10 mg/kg induced; Group C30: Doxycycline 30 mg/kg induced; Group Se: Sedentary control; Se10 Group: Sedentary 10 mg/kg/day; Se30 Group: sedentary lifestyle 30 mg/kg/day; OVX Group: control Ovariectomized; OVX10 Group: Ovariectomy 10 mg/kg/day and OVX30 Group: Ovariectomy 30 mg/kg/day.

Figures 7 and 8 shows histological images of cancellous bone with the thickness of 5 µm on the area of 30 mm², the presented results was expressed in Pixel^2^ ^22,23^ by the software AxioVision 4.8 (Carl Zeiss, Oberkochen, Germany) which distingue the cancellous bone from cortical bone and the growth plate portion, using the spectrum of color to delimitated the area of interest.

Figure 7C presented a mineralized normal bone tissue with cancellous bone distributed uniformly (Cn) and the growth plate across the distal portion of the bone as normal and uniform mode (Gp). Figure 7 -C10 had a mineralized bone tissue with the same distributions, however to a lesser amount of cancellous bone when comparing with the bone tissue of Fig. 7C as the cancellous bone (Cn) appeared slightly more spaced. Growth plate (Gp) showed ticker aspect in this section. Figure 7 C30 had the presence of a mineralized bone tissue with slight decreases in bone quality. This fact may be caused by the reduced amount of cancellous bone (Cn) and wide space distance between cancellous tissue contrasting with the control (Fig. 7C).

Figure 7 -Se showed the presence of a bone tissue affected by a degenerative disease caused by sedentary Wistar rats induced in females. Cancellous bone (Cn) showed a large spacing between themselves and the growth plates (Gp) altered more their thicknesses. Figure 7 -Se10 showed a great improvement of the cancellous bone tissue (Cn) combined with an irregularity presented in growth plate (Gp) demonstrating that doxycycline is able to change the growth plate morphology. Figure 7 -Se30 demonstrated a large amount of cancellous bone tissue (Cn) and a change more evident in the growth plate (Gp).

Figure 7 -OVX had the presence of a bone tissue affected by a degenerative disease caused by bilateral ovariectomy in female Wistar rats, with spacing of cancellous bone (Cn) and thick growth plate (Gp) (Yao *et al*., 2006). Figure7 -OVX10 and -OVX30 had been an increase in the number of cancellous bone and a decrease in the changes in growth plate thickness (Gp).

Figure 8 (proximal femur) showed no significant differences in cancellous bone (Cn), in exception for OVX group. It was found changes in growth plate (Gp) on samples of rats exposed to Doxycycline for 60 days (Figures-C10, C30, Se10, Se30 and OVX30 and this growth plate was absent completely in Figure-OVX10). According to statistical differences found in Table 2, OVX vs. OVX10 p = 0.04 and OVX vs. OVX30 p = 0.05 had statistically significant.

#### Pricrosirius-red

Data obtained from samples submitted to picrosirius red staining (data not shown) did not shown statistical differences on total collagen (Table 2). It suggests that the doses were not enough for changing the total amount of total collagen on bone, but it well known that the inhibition of collagenase is one of the actions of doxycycline^57–59^. This fact did not result in changes on the quantification of total collagen at this work.

## Discussion

Data of immunolabeling for TRAP showed semi-quantitative differences, with higher immunostaining identification for control group compared to the exposed to doxycycline 10 mg/kg/day and 30 mg/kg/day, demonstrating that the doxycycline in these two doses inhibited osteoclast action in this study. This type of inhibition had been described in previous *in vitro* studies^60,61^.

Figure 1- shows the molecule of doxycycline^62,63^ in the anhydrous form (Fig. 1). The lower portion of the molecule doxycycline is capable of bivalent metal binding^1^. A study affirmed that in the presence of Zn^2+^ ions, there is the occurrence of competition for the catalytic site of MMP-7 connected prior to Ca^2+^ Ion, thus inhibiting activity of MMP-7^64^, although it is known that tetracyclines are chelators of Zn^2+^ ions^65^, the actual mechanism of inhibition of MMPs are not yet clear.

Tetracyclines have 4 aromatic rings (Fig. 1) and also have characteristic of chelating bivalent metal in physiological conditions, forming chelate complexes^66^. In this study, the authors tested the association (calculating the equilibrium constants of association) between several divalent metals and tetracycline, considering the following metals: Mg, Ca, Sr, Ba, Mn, Fe, Co, Ni, Cu, Zn and Cd. The constant of association varied in 3 orders of magnitude, and the results show that under physiological conditions, the metals with higher-to-low affinity for tetracycline are first order Ba, Sr, Cd, followed by the second order Ca, Mg, Cu, Mn and Zn. Only the third order Fe, Ni and Co had lower affinity than zinc (about 5 times less). However, since the physiological levels of calcium are at the millimolar level, which is the metal that is connected to tetracycline. Other divalent metals are in much lower concentrations^66^.

In addition, tetracyclines inhibit MMP-13 and this type of MMP is intrinsically related to the biology of growth plate of the bones. The type X collagen is find in smaller quantities in MMP-13 knockout mice and the lack of expression of this type of collagen are related to the dwarfism and is related to changes in activities of chondrocytes^67^. It is known that the hypertrophic chondrocyte is responsible for three actions on bone: 60% of bone growth, 30% of the mineral deposition in the bone and 10% of cellular proliferation^68^.

MMP-2 was first isolated from bone, and it is very important for normal bone maintenance^69^. Lack of activity of MMP-14 (MT1-MMP) induces dwarfism, osteopenia, arthritis, and connective tissue disease^70^. Therefore, the use of doxycycline would also inhibit MT1-MMP and other MMPs^71^, since this drug is not selective.

Morphological analysis showed a clear improvement on the quality of cancellous bone accordingly to histological analyses (Figs 7 and 8). Data from distal portion of the femur bone in Wistar rats with osteopenia induced exposed to two doses of doxycycline (Se10, Se30, OVX10 and OVX30), permits to infer that this medicine is able to improve the quality of the bone structure compared to the control groups (C, Se and OVX). This finding was described in Table 2 and Fig. 7 and is supported by another study using orthodontic tooth movement in mice^72^. On this related study there was a smaller dental root resorption in rats subjected to Doxycycline for 7, 10 and 14 days of orthodontic treatment and on orthodontic movement it was found lesser amount for TRAP immunostaining at the group exposed to Doxycycline during tooth movement^72^. Gomes and Fernandes concluded an *in vitro* study that doxycycline and minocycline promoted human osteoblastic cells proliferation and this leaded to a later mineralization, which allowed to consider that doxycycline may induce bone mineralization^73^ and less bone resorption^72^. Considering this kind of dental intervention it is well sedimented in the literature that orthodontic movement induces an inflammatory reaction, which is considered a normal side effect for orthodontic practice^74^.

Limirio and collaborators showed that doxycycline 10% associate to alendronate 1% increase the time of bone neoformation on 7 and 15 days on rats with femur osteotomy. Their findings suggests that doxycycline associated to alendronate on the defect suppress the osteoclasts and had less bone neoformated on 7 days^75^, this data corroborate to our findings on controls groups (C, C10 and C30), which doxycycline SDD have a negative rule on the physiological bone. But long-term treatment (60 days) of SDD on physiological bone, without osteopenia, was never tested. Normally the doxycycline is studied for bone repair or alveolar repair^76^ with positive results, but never tested on long-term SDD on long bones.

Another study performed in dogs, showed that doxycycline is able to reduce bone loss caused by endodontic peri radicular surgery^77^. However, another study showed that doxycycline does not improved the quantity and quality of tissue repair on bone in Wistar rats subjected to dental extraction and subsequently treated for 7 days on doxycycline and erythromycin^78^. This fact may be comprehended in this previous cited work, explaining that the rats were subjected to a short period of treatment with doxycycline (7 days) and the quantification was injured because of this short time of treatment.

In this present research, doxycycline inhibited osteoclastic bone action^76^, and therefore this drug was beneficial for bone under conditions of high osteoclastic activity. However, under normal physiological conditions, it is not beneficial for bone. It is worth pointing out that doxycycline can increase osteoblast action, perhaps by modifying the unbalance caused by osteoporosis where it had an increase in the action of osteoclasts cells, and a decrease in the action of osteoblasts cells^18,76^. Apparently looking for this topic at current literature and results contained in this present work, we can summarize that doxycycline decreases osteoclastic action^60,61,76^ and increases osteoblast action^73,76^. However, it is not known how this “re-balance” happens, further studies are needed to prove that.

It is well known that doxycycline chelates divalent metals^66^ and this interaction between doxycycline and divalent metal calcium is important for the health of bone tissue, as in the study of Weaver and colleagues sought to measure bone quality via the urinary excretion of Calcium isotopes. The authors used an isotope of tetracycline to make this measurement and with that, the authors could measure almost immediately the differences after induction of osteopenia by ovariectomy^79^.

There are several questionings about the fact that doxycycline being an antibiotic and does not present an increase of microbial resistance. In humans, the dose of 20 mg/kg/day does not presented increased subgingival flora^80^ and it is used as metalloproteinase inhibitor for treatment of periodontitis^5,81^. Furthermore, the use of doxycycline at this dose has already been proven not antimicrobial (SDD), not affecting the skin floras^82^. As the tetracyclines, minocycline and doxycycline, are widely used for antimicrobial purpose (skin problems) at SDD^83–88^.

Data shows that the amount of calcium present in serum seemed to had contributed positively for bone increase (with an increase in serum calcium of samples from the OVX10 and OVX30 groups compared with OVX). Densitometric and histological findings corroborate with the clinical improvement of bone quality. Calcium homeostasis is a mechanism closely regulated in our body^89^, so, further studies are needed to determine whether doxycycline really changes serum calcium and whether those changes reflect bone changes or induce bone changes. It is tempting to say that doxycycline can carry calcium to the bone in osteoporotic rats. The same affirmation is not true with controls, in which doxycycline had deleterious effects.

In conclusion, SDDs for a long time (sixty days) in 2 models of osteopenia (hormone-dependent and sedentarism-induced) in female rats had positive effects on bone, particularly well characterized the decrease in osteoclastic activity. On the control groups doxycycline were mostly deleterious, with exception the quantification of cancellous bone with a slight increase of collagen quantification on proximal portion of the femur. Osteopenic animals also showed increased densitometric data in bone analyses in femurs, and a higher quality of cancellous bone was observed in the osteopenic groups that received doxycycline. According to the results of this study, doxycycline had a high therapeutic potential for the long-term treatment of osteopenia.

## Supplementary information

Supplemental information file 1
