# Supplementary material for: Doxycycline reduces osteopenia in female rats
Source: Sci Rep. 2019 Oct 25;9:15316. doi: 10.1038/s41598-019-51702-y (PMC6814740; doi:10.1038/s41598-019-51702-y)

**Doxycycline reduces osteopenia in female rats**

Fellipe A. T. de Figueiredo ^1^

Roberta C. Shimano ^1^

Edilson Ervolino ^2^

Dimitrius L. Pitol ^3^

Raquel F. Gerlach ^3^

Joao Paulo M. Issa ^1,3^

¹ Department of Biomechanics, Medicine and Rehabilitation of the Locomotor System, Ribeirao Preto Medical School, University of Sao Paulo – Av. Dos Bandeirantes 3900, Ribeirao Preto, SP, Brazil. CEP 14049-900.

² Department of Basic Sciences, Sao Paulo State University Júlio de Mesquita Filho – R. Jose Bonifácio 1193, Araçatuba, SP, Brazil. CEP: 16015-050.

³ Department of Morphology, Physiology and Basic Pathology, School of Dentistry of Ribeirao Preto, University of Sao Paulo – Av. Cafe S/N, Ribeirao Preto, SP, Brazil. CEP: 14040-904. +55 16 33154094

**Corresponding author:** Prof. Dr. Joao Paulo Mardegan Issa

School of Dentistry of Ribeirao Preto, University of Sao Paulo, Brazil

Av. Cafe S/N-Ribeirao Preto-SP, Brazil. CEP: 14040-904

Tel: +55 16 33159136 Fax: +55 16 3315-4102

email: jpmissa@forp.usp.br

Supplemental information file 1: Flow chart with the bone biopsy method explained.


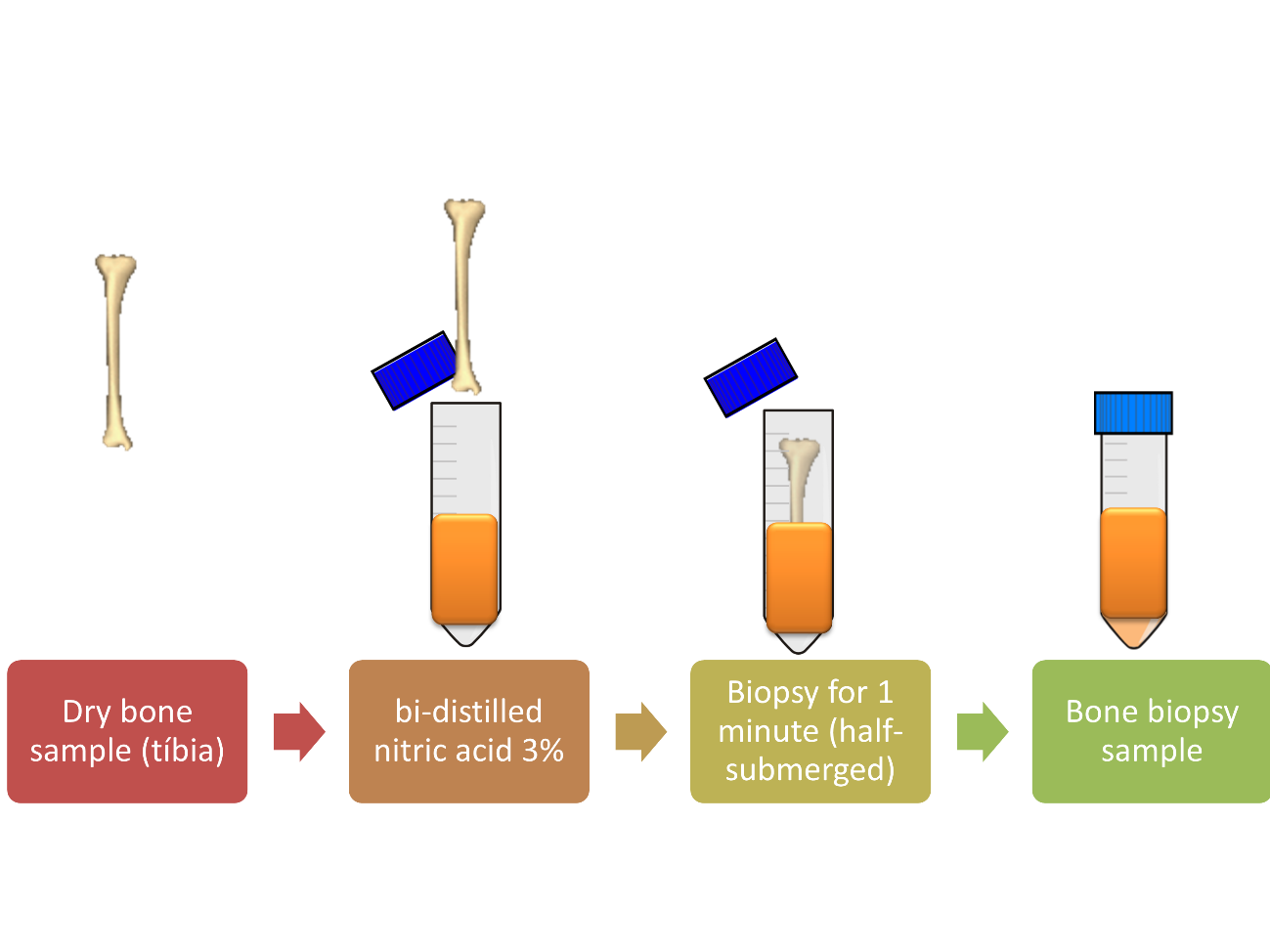

Supplement: Supplementary file 1 — Supplemental information file 1 [file 41598_2019_51702_MOESM1_ESM.docx]
